# Socially assistive walker for daily living assistance in older adults

**DOI:** 10.3389/frobt.2024.1401663

**Published:** 2024-08-16

**Authors:** Sergio D. Sierra M., Nigel Harris, Marcela Múnera, Carlos A. Cifuentes

**Affiliations:** ^1^ Faculty of Engineering, University of Bristol, Bristol, United Kingdom; ^2^ Bristol Robotics Laboratory, University of the West of England, Bristol, United Kingdom

**Keywords:** socially assistive walker, ageing population, healthcare robotics, activities of daily living, user preference

## Abstract

**Introduction:**

This paper addresses the growing healthcare needs of an ageing population and the lack of advanced technologies with social capabilities that are cost effective, user friendly, and readily adopted. In response to this motivation, a socially assistive walker is designed to provide physical and cognitive support in activities of daily living for older adults.

**Methods:**

Physical and cognitive support is provided by walker’s structure, sensors, and feedback interfaces to assist users daily living activities, as well as, in navigating environment safely and efficiently. The walker’s design arises from semi-structured interviews conducted with ageing experts, leading to the development of two levels or modes of social interaction, namely low and high interaction.

**Results and discussion:**

In a cohort of 14 adults, the study found the device easy to use regardless of the interaction mode, with 78.5% expressing a preference for the version featuring embodiment, verbal feedback, and more proactive cues (*p* < 0.05). The results also prompted ideas and suggestions for new designs based on insights gleaned from the user. This research contributes to the field of socially assistive robotics by offering an example of a user centred approach to address the healthcare challenges an ageing population poses.

## 1 Introduction

The landscape of global demographics places an increasing emphasis on providing healthcare and social services for older adults, aiming to ensure their quality of life and overall wellbeing (United Nations and Social Affairs, 2020). In the UK, this demographic shift is notably evident, with approximately 11.6 million individuals aged 65 or older, projected to increase an additional 8.6 million by the year 2075 (Office for National Statistics, 2018). Ageing introduces challenges, including hearing loss, vision impairments, musculoskeletal disorders, depression, and dementia (Maresova et al., 2019). These age-related changes can significantly impact an individual’s independence, often leading to reduced ability to perform daily activities and maintain social interactions. Consequently, this loss of independence can contribute to social exclusion, as older adults may withdraw from community engagement and experience decreased social support (Donovan and Blazer, 2020). The combination of physical limitations and social isolation can severely affect the quality of life, leading to feelings of loneliness, reduced mental health, and overall diminished wellbeing. In this sense, ^R1^priority areas for developing assistive technologies for older adults include gait disorders and falls, support for cognitive function and mental health, and promoting social inclusion.

The ageing process affects mobility and balance, resulting in deviations from the natural gait pattern and a decline in its quality (Auvinet et al., 2017). These age-related disorders not only compromise physical wellbeing but also significantly elevate the risk of falls, potentially causing more injuries and further impairment (Auvinet et al., 2017). A substantial 40% of older adults living in their homes experience at least one fall annually, with even higher rates in care homes, translating to a cost estimate of £2.3 billion per year. As a response, many older adults turn to walkers, with 22% utilising them indoors and 44% outdoors (Thies et al., 2020). However, while aiding mobility, walker frames present heightened cognitive demands for both cognitively healthy older adults and those with cognitive impairments. Particularly when navigating complex paths and manoeuvring around obstacles, paradoxically, walker usage increases the risk of falling and might lead to tripping incidents and disruptions in balance control (Pirker and Katzenschlager, 2017; Hunter et al., 2019).

Furthermore, the ageing process is accompanied by declines in specific cognitive abilities, primarily associated with executive functions (higher-level planning and problem-solving), attention, and episodic memory (Veríssimo et al., 2021). Atypical ageing, including anxiety, stress, depression, mild cognitive impairment and dementia, brings more pronounced cognitive decline. Notably, the prevalence of dementia is on the rise, with around 900k people currently living with dementia in the UK, projected to exceed 1.6 million by 2050 (UK and Hub, 2022).

In response to these challenges, the past two decades have seen active research into assistive robots, encompassing social robots, wearable devices, and smart walkers, designed to support older adults dealing with gait disorders, fall prevention, and cognitive impairments (Łukasik et al., 2020; Soufineyestani et al., 2021). However, despite these efforts, achieving widespread user adoption remains an ongoing challenge due to factors such as multi-targeted assistance (i.e., physical and cognitive), costs, and usability (Getson and Nejat, 2022; Medrano et al., 2023). For instance, in terms of multimodal assistance, social robots have proven to be efficient in providing companionship to older adults (Hirt et al., 2021), while robotic walkers exhibit a high potential for physical assistance (Sierra M. et al., 2021). However, individually, these devices provide opportunities to deliver physical support to users during daily living activities (i.e., social robots) and to motivate users through friendly, intuitive, and personalised interaction (i.e., robotic walkers). P

Recent research underscores the necessity of integrating the strengths of both social robots and robotic walkers to develop a multimodal assistive device that provides comprehensive support for older adults. Such a device would not only offer physical assistance but also engage users through social interaction, addressing both mobility and social wellbeing (Sierra M. et al., 2021; Zhao et al., 2020; Sierra M. et al., 2019; Luz et al., 2015; Bardaro et al., 2022; Stegner and Mutlu, 2022; Lundberg et al., 2022; Nauta et al., 2019).^R1^

This work presents the design of a social smart walker as the result of a collaborative approach with healthcare experts. This device integrates proven and cost-effective technologies from socially and physically assistive robots into the conventional walker frame. Specifically, this research explores the integration of a social agent into a mobility aid tailored for older adults, specifically a conventional rollator or walker. This work encompasses expert-based design criteria for a social smart walker and its preliminary validation using an experimental assessment involving two distinct social interaction levels for daily living assistance in adults. The proposed validation study aims at addressing the research question: *How to maximise the acceptance and adoption of a robotic walker during daily living assistance in older adults employing multimodal levels of social engagement?*

## 2 Related works

Multiple types of robots, including social robots, wearables, and smart or robotic walkers, have addressed daily living assistance. Social robots are designed to provide companionship and support through tasks such as medication reminders, health status monitoring, and emotional support (Céspedes et al., 2021; Getson and Nejat, 2022). However, they face multiple adoption barriers, encompassing relatively high costs compared to alternative technologies (Moyle et al., 2014; Gross et al., 2015), as well as constrained adaptability due to complex pre-programmed functions and user interfaces (Bajones et al., 2018). Similarly, other factors affect the users’ perception and adoption, such as cultural paradigms, limited user familiarity with technology (especially among older adults), and methodological challenges in qualitative studies (Woods et al., 2021). In this sense, research involving commercial robots has underscored the need for more extended interactions, real-world testing, and early-stage collaboration with stakeholders to enhance acceptability (Alonso et al., 2019; Bradwell et al., 2019; Rico et al., 2020; Dosso et al., 2022).

Robotic walkers aim to address gait disorders by incorporating sensors and advanced algorithms to provide tailored assistance to users (Sierra M. et al., 2021, Sierra M. et al., 2022). These devices offer partial weight support while also detecting potential falls and mechanical stabilization with feedback on gait patterns and navigational guidance (Ding et al., 2022). Additionally, a recent review (Verdezoto et al., 2022) analysed multiple studies on smart rollators, focusing on testing, interfaces, and control modes. Most studies have not tested rollators with the target population, relying instead on healthy volunteers. Validated systems, tested by elderly or specific disease populations, typically use force sensors for gait and support monitoring. Rollator interfaces are non-invasive, primarily using upper limb interactions with handlebar sensors like force and torque sensors. Feedback is provided through haptic and visual indicators, with some systems incorporating additional sensors like IMUs and cameras. Recent rollators have shifted to passive or shared-control modes to avoid balance issues. They monitor user conditions and provide assistance with steering, collision avoidance, and energy-saving functions (Verdezoto et al., 2022).^R2^ Nevertheless, adopting smart walkers remains constrained by factors such as cost, user-friendliness, social presence, and the complexity of functionalities (Ferrari et al., 2020; Werner et al., 2020; Sierra M. et al., 2019; Guamushig-Laica et al., 2022).

In this scenario, there is a need to develop a multimodal assistive device that brings together the benefits of social robots and the physical support and monitoring abilities of robotic walkers. Particularly, literature evidence suggests that robotic walkers can assist with activities of daily living (Sierra M. et al., 2021), prevent falls (Zhao et al., 2020), and monitor physical activity (Sierra Marín et al., 2019). However, similar to conventional walkers, older adults tend not to use their devices at home, which is common when falls occur (Luz et al., 2015). Reasons often include forgetfulness, negative feelings of ageing evoked from the device, and inaccessibility, among others (Luz et al., 2015). Alternatively, social robotics, while they lack physical support, offer a great potential to engage with users (Céspedes et al., 2021), enable more intuitive interaction, and provide companionship (Bardaro et al., 2022). In this sense, designing for caregiving in older adults demands physical support, cognitive support, diverse interaction, and communication (Stegner and Mutlu, 2022). Some recent approaches have targeted such multimodal interaction by integrating service robots to walkers as robotic nursing assistants (Lundberg et al., 2022) or mounting social robots into mobile robots to assist multiple users and proactively position the robot in care homes (Nauta et al., 2019). However, these studies still report systems’ complexity negatively affecting user adoption, expensive robotic platforms, and, in some cases, lack of actual physical support.

## 3 Methodology

### 3.1 Platform design

The robotic platform presented in this work results from evidence from literature and the outputs of semi-structured interviews with healthcare experts. These information sources helped to identify the functionalities and design requirements for a Socially Assistive Walker (SAW) that is capable of providing daily living assistance to older adults while maintaining both physical and social abilities. The inclusion of clinicians’ views in the design phase is essential for several reasons. Clinicians possess a deep understanding of the day-to-day challenges faced by older adults, particularly those related to mobility, social interaction, and overall health management. Their expertise ensures that the design of the SAW addresses real-world needs and integrates seamlessly into the existing care routines. Moreover, clinicians can provide valuable feedback on the practical aspects of usability, potential barriers to adoption, and the anticipated impact of the technology on patients’ quality of life.^R1^

Specifically, a group of geriatric care experts was formed by 14 professionals from the England NHS in partnership with AgeUK, Gloucestershire, a neuro-physiologist from CIUSSS West-Central Montreal, an expert consultant former Chief Executive of a UK assistive technology institution, and a senior professor in dementia research from the University of the West of England.

**FIGURE 1:**
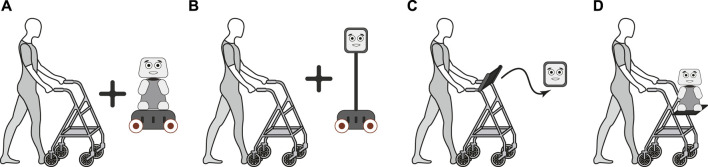
Design alternatives for a socially assistive walker. **(A)** Walker and social robot on a mobile platform. **(B)** Walker and telepresence robot. **(C)** Walker and tablet with a virtual agent. **(D)** Walker with a social robot on top.

Semi-structured interviews were conducted with a group of experts to extract the design criteria of the SAW. Initially, a set of design alternatives was introduced to the healthcare experts, specifically, four design alternatives integrating social abilities into a walker frame (See Figure 1). The experts were asked to comment on the expected features that should be integrated into the robotic platform, the barriers of existing similar technologies, the expected usability, and the potential applications of this kind of technology.

The categories used for grouping the experts’ comments included: 1) Physical Support, the ability to provide robust physical assistance, especially for users with mobility impairments; 2) Cognitive Support, features that support cognitive functions, including reminders and interaction prompts; 3) Tasks Support, assistance with daily tasks and activities of daily living (ADLs), 4) Appearance, aesthetic considerations to ensure the device is appealing, and 5) Safety, ensuring the device is safe for daily use, minimising risks of accidents or malfunctions. Moreover, clinicians were asked to sort the design alternatives based on the following considerations: outdoor usability, technological complexity, and cost.^R1^

In general terms, the outcomes of the interviews, as a first approach to healthcare institutions, revealed that a robotic walker with social abilities could be a potential solution to mitigate falls in older adults, which is the predominant factor of hospital bed occupancy outlined by the local NHS care board. Furthermore, deploying an SAW could help reduce the workload of caregivers and clinicians, as it can monitor the health state of the users and assist them in activities of daily living. Likewise, experts from care homes stated that deploying a SAW in assisted living homes and residential scenarios will reduce the burden on healthcare systems and allow better on-site and remote access. Table 1 highlights the main design criteria and expected features extracted from the literature and the semi-structured interviews.

**TABLE 1:** Summary of design criteria for a socially assistive walker.

Theme	Criteria / Features
Physical Support	- Gait support and partial weigh bearing.
	- Posture tracking.
	- Activity monitoring.
Cognitive Support	- Natural and multimodal feedback.
	- Companionship.
	- Coaching and motivation.
	- Adaptive interaction levels.
	- Personalised interaction.
Tasks Support	- Sit-to-stand and stand-to-sit transfers.
	- Food and medication reminders
	- Companionship during walking.
	- Indoor guidance.
	- Retain frame seating option.
Appearance	- Avoid over-engineered appearance.
	- Reduce electronics visibility
	- Retain mechanical frame
Safety	- Retain frame brakes.
	- Provide additional automatic brakes.
	- Ensure front-facing focus.

Key articles that significantly influenced the design criteria and features are summarised as follows. (Céspedes et al., 2021; Dosso et al., 2023). focuses on social robots contributing to the criteria related to companionship, emotional support, coaching and motivation. (Sierra M. et al., 2019; Sierra M. et al., 2021; Piau et al., 2019). focuses on robotic walkers contributing to the criteria related to gait support, activity monitoring, and tasks support. (Belpaeme et al., 2018; Rubagotti et al., 2022; Leonardsen et al., 2023). contributed to criteria related to appearance and safety.^R1^

Regarding the interviews, they were coded using thematic analysis. Themes were grouped into the categories presented in Table 1, reflecting the main design criteria and expected features.^R1^

According to the above, and considering the experts’ comments on keeping outdoor usability, avoiding technological complexity, and ensuring low-cost alternatives, the third design alternative was selected (See Figure 1C). Moreover, to meet the criteria presented in Table 1, as well as the ability to daily living assistance, several sensors and feedback interfaces were selected.

These articles provided insights into the technological advancements, user needs, and challenges associated with current assistive devices for older adults.

### 3.2 Socially assistive walker (SAW)

**FIGURE 2:**
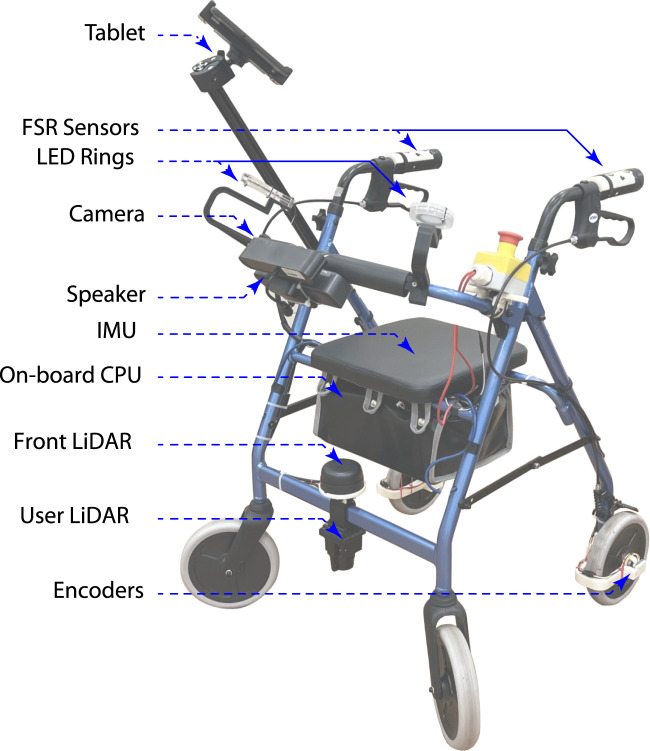
Illustration of the Socially Assistive Walker (SAW) and the feedback interfaces and sensors attached to it.

A SAW was designed for this work as a conventional walker frame equipped with multiple sensors and feedback components to provide visual, auditory, and user interface capabilities. Figure 2 shows the designed platform with its sensors and feedback interfaces.

The SAW equips a Raspberry Pi 4–8 GB (Raspberry Pi Foundation, UK) that runs the Robotic Operating System (ROS) on top of a Linux distribution. A pair of magnetic encoders (AS5600 Magnetic, Osram, Germany) were integrated into the rear wheels, as well as an Inertial Measurement Unit (IMU) (BNO080, SparkFun, United States) below the frame seat to estimate the walker’s odometry. A front LiDAR (RPLIDAR A3 360, Slamtec, China) was placed on the frame to assess obstacles and provide mapping. A rear LRF (URG-04LX-UG01, Hokuyo, Japan) was placed on the frame to track the user’s legs. A USB speaker (HONKYOB, China) was integrated into the platform for auditory feedback. A USB camera (Astra, Orbbec, China) was incorporated and its colour stream is used for posture tracking. A pair of LED rings (NeoPixel x16, Adafruit, United States) were integrated for visual feedback. A pair of FSRs (402, Interlink Electronics, United States) were placed on the handles to track gripping pressure. And finally, a tablet (Galaxy Tab A, Samsumg, South Korea) is attached with a custom extender and holder on the side.

An additional external computer was used to off-load processing pressure from the onboard microcomputer, record experimental data, and serve as a web-socket server for the tablet. This external computer also served as the workstation to monitor the sensors and trigger feedback behaviours on the device.

### 3.3 Multimodal social engagement: case study

To assess the acceptance of the proposed SAW, we developed two interaction strategies or social engagement levels for assistance during daily living activities.

#### 3.3.1 Interaction strategies

According to literature, different levels of social interaction can be achieved by modulating the information provided by the robot, the non-verbal and verbal cues employed to communicate information, the use of dialogues, and the level of embodiment (Onyeulo and Gandhi, 2020; Paetzel et al., 2020; Tan et al., 2020). In this sense, the designed interaction levels were classified as high and low-engagement strategies and were configured as follows:

##### 3.3.1.1 High interaction

The SAW was endowed with embodiment through the presence of a virtual agent displayed on the device’s tablet. The agent’s design leans toward a more robot-like appearance to mitigate potential issues related to deception and ethical concerns stemming from the human-like effect and anthropomorphism (Paetzel et al., 2020; Winkle et al., 2021). Nevertheless, to preserve a sense of natural interaction and a minimal level of perceived vitality, the agent has been programmed to engage in sporadic blinking, subtle size fluctuations to mimic motion, and mouth movements while communicating. Regarding feedback, the system employs LED rings to convey a wide range of interactions and states. When tasks are completed, the LED rings emit a reassuring green colour. In situations requiring caution or while waiting for user input, the yellow colour signals this state. A red colour indicates errors. During processing, the LED rings smoothly transition through various shades of blue. When the system is guiding the user, a flashing blue colour is utilised, which can appear on either one side or both sides simultaneously. To further enrich the user experience, auditory feedback is incorporated, utilising Google’s text-to-speech API for generating voice commands. Furthermore, to facilitate personalised and proactive communication, the robot employs the user’s name as an initial point of interaction and explains the available feedback interfaces. Positive feedback is delivered with the inclusion of supportive adjectives, and clear yet comprehensive instructions are provided to enhance the user experience.

##### 3.3.1.2 Low Interaction

In this mode, the SAW operates without embodiment, and consequently, the virtual agent is omitted. Instead, the device’s tablet displays straightforward text instructions, adjusted to a comfortable font size for the user. Feedback is conveyed through the same LED ring states, and notification sounds are employed to signal the presence of new information on the tablet. In this context, instructions are intentionally kept concise and straightforward, omitting the use of the user’s name and additional supportive adjectives to simplify the interaction.

#### 3.3.2 Feedback during activities of daily living

A selection of daily living activities was chosen: 1) greetings, 2) stand-to-sit transfers, 3) reminders for food and hydration, 4) sit-to-stand transfers, 5) indoor guidance, and 6) 2 min of walking. The SAW was programmed to gather data from onboard sensors and deliver suitable feedback during these activities. To accomplish this, the SAW was equipped with additional software modules, such as 1) the ROS navigation stack with a pre-defined map to track the user’s movement within the experimental environment and 2) Google’s Mediapipe library to estimate the user’s posture and sitting state (Google Developers Team, 2023). For safety considerations, an external operator closely monitored and remotely initiated feedback interventions using the information provided by the sensors and the device’s software modules in all cases.

Table 2 describes the system states, the given information, and the feedback provided by the system. In all cases, the operator’s task was to control the flow of the system states by checking whether the users followed or ignored the robot’s feedback. When the user followed the instruction, either positive feedback was given with “*Great job!”* or “*Great*” cues for high and low interaction, respectively. Instructions were only given once, and no re-forcing or repetitive strategy was programmed. In these cases, a 5-second timeout was considered to go to the next state. In the case of the guidance activity, positive feedback was only given when the user reached the kitchen, bathroom, and living room inside the experimental studio.

**TABLE 2:** Description of utterances under high and low interaction modes, including system states, and lights behaviour.

Mode	System State	Information	Lights
High	Greeting	Hello <name>, I am your walker buddy, here to assist you with tasks today. I will talk to you and use lights and my screen.	Green
	Stand-to-Sit	1. Approach the chair next to the nightstand. Wait standing in front of the chair, with the back of your legs touching the front edge of the seat [Wait for user]	Yellow
		2. Keep your feet shoulder-width apart for stability. Engage my brakes and maintain a relaxed but secure grip. [Wait for user]	Yellow
		3. Slowly begin to lower yourself into the chair by bending your knees. Use your legs muscles to control the movement. [Wait for user]	Yellow
		4. Take a moment to adjust your position and ensure you are stable and comfortable in the chair. You can use the armrests for extra support. [Wait for user]	Green
		5. Great job! Hope you enjoy your resting time.	Green
	Food reminder	1. Having a balanced and nutritious meal is great to keep you energised and healthy.	Green
		2. In the table next to you, there are some fruits over there. Pick one and enjoy it! [Wait for user]	Blue tones
		3. Drinking water throughout the day is also excellent for your health. Drink some from the glass on the table! [Wait for user]	Blue
		4. Well done! You’ve completed your food reminder. I am here to support you every step of the way!	Green
	Sit-to-Stand	1. Please engage my brakes. To do so, push them down or compress them. [Wait for user]	Yellow
		2. While firmly holding both handles, stand up keeping your back straight. [Wait for user]	Yellow
		3. Nailed it. Now you can release the brakes!	Green
	Guidance	1. Alright, let’s move to different rooms. I’ll provide you with easy directional cues to guide you.	Green
		Turn left. [Wait for user]	Left blue
		Turn right. [Wait for user]	Right blue
		Walk forward. [Wait for user]	Blue
		Turn around. [Wait for user]	Yellow
		Done! Take a moment to relax and rest	Green
	Walking	1. Walking is an excellent wait to stay active and healthy	Green
		2. Stand tall and hold onto the hand-grips with both hands securely. Ensure brakes are disengaged. [Wait for user]	Yellow
		3. Start walking at a comfortable pace. Take small, steady steps while keeping your back straight and looking forward.	Blue tones
		[30 secs] You’re doing great, you’re almost there!	Green
		[30 secs] Walking helps you stay healthy, we are halfway there.	Green
		4. Awesome! You’ve completed 2 min of walking. Slow down gradually and come to a stop	Yellow
	Goodbye	Awesome. You’re all set for today. Great job!	Green
Low	Greeting	Hello, I will assist you today.	Green
	Stand-to-Sit	1. Go to the chair next to the nightstand. Stand with the back of legs touching the front edge of the seat. [Wait for user]	Yellow
		2. Keep your feet apart. Engage brakes. [Wait for user]	Yellow
		3. Begin to lower yourself into the chair by bending your knees. [Wait for user]	Yellow
		4. Adjust your position in the chair and this task is done.	Green
	Food reminder	1. Nutritious meals are important for health.	Green
		2. In the table, there are fruits. Pick one.	Blue tones
		3. Drinking water is also important. Drink some.	Blue
		4. This task is done.	Green
	Sit-to-Stand	1. Engage the brakes. [Wait for user]	Yellow
		2. Keep them engaged. Hold both handles, and stand up with your back straight. [Wait for user]	Yellow
		3. Great. Now release the brakes	Green
	Guidance	1. First go to the kitchen. [Wait for user]	Green
		*Same as high interaction mode.*	*Same*
	Walking	1. Now we will prepare to walk for 2 min.	Green
		2. Stand straight and hold both handles. Brakes off.	Yellow
		3. Start walking. Take steady steps and look forward. [60 secs] Keep going.	Blue tones
		4. Done. Slow down and come to a stop.	Green
	Goodbye	Tasks completed. Goodbye	Green

### 3.4 Experimental setup

#### 3.4.1 Participant recruitment

Healthy adult subjects participated in the study. Participants eligible for the study were adults aged 40 years or older who possess at least a minimal level of familiarity with assistive technologies, can provide informed consent themselves, and have sufficient cognitive abilities to comprehend and adhere to study instructions. Excluded from participation were adults with severe cognitive impairments or dementia that hindered their ability to engage in the study, individuals with uncontrolled medical conditions, those with severe visual or hearing impairments, and individuals with a history of falls or balance issues.

#### 3.4.2 Session procedure

The sessions occurred at the Assistive Living Studio of the Bristol Robotics Laboratory at the University of the West of England. Each participant was asked to attend one session. The studio resembles a house with a living room, kitchen, bedroom, and bathroom. Each session consisted of two trials, one for each level of social engagement. At the beginning of the session, a demographics questionnaire was applied, and a brief explanation of the activities was given without giving particular details on the levels of interaction. The participants were asked to start each trial with their hands on the handles and standing straight. The researcher also asked the participants to pay close attention to the instructions the walker gave, and they were not informed that an external researcher was monitoring and controlling the interaction. At the end of each trial, the participants were asked to fill out a perception questionnaire, and at the end of the session, an open-ended questionnaire was applied.

#### 3.4.3 Qualitative assessment

The demographics questionnaire aimed to uncover participants’ prior experiences and current usage of conventional and robotic assistive devices. It also assessed their current living arrangements and the frequency of their daily interactions with others.

The usability questionnaire was based on UTAUT surveys and previously validated perception surveys for social robots (Heerink et al., 2009; Venkatesh et al., 2012; Horstmann and Krämer, 2020), to evaluate perceptions of each level of social engagement. This assessment encompassed six distinct categories: 1) Anxiety (ANX) to measure concerns about using or potentially damaging the device, 2) Attitude (ATT) to measure participants’ willingness to incorporate the device into their current or future daily activities, 3) Facilitating Conditions (FC) to identify comprehension of the interaction strategy, 4) Perceived Enjoyment (PENJ) to assess preferences regarding the interaction strategy, 5) Perceived Ease of Use (PEOU) to evaluate intuitiveness, complexity, and communication, and 6) Social Presence (SP) to analyse social interaction skills, perceived intelligence, and engagement preferences. Responses to the questions were asked using a 5-point Likert scale ranging from “*completely disagreed*” to “*completely agreed*”. Statistical analysis employed the Mann–Wilcoxon (MWW) test to determine the presence of significant differences between high and low levels of interaction^R1^.

Lastly, the open-ended questionnaire aimed to identify participants’ overall preferences for a specific level of social engagement and gather impressions regarding missing features, features to eliminate, and features requiring modification.

#### 3.4.4 Ethical considerations

The experimental protocol was registered with the University’s ethical committee and approved in a timely manner. All participants read and signed the informed consent document. After sessions, videos/data were removed from the external camera used to record them and uploaded to a safe storage location in the University’s cloud drive, following the University’s data protection guidelines.

## 4 Results and discussion

**FIGURE 3:**
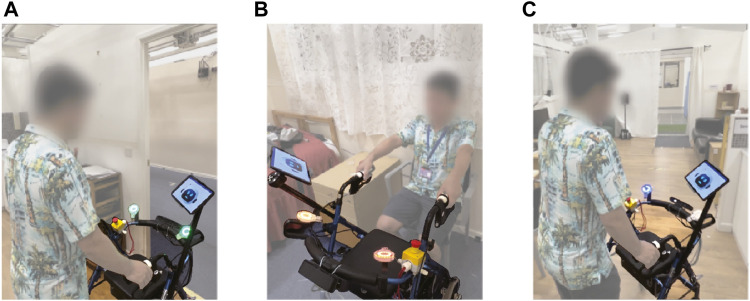
Experiment illustration. **(A)** Smart walker during greeting and ready state. **(B)** Smart walker assisting sit-to-stand. **(C)** Smart walker providing indoor guidance with left LED.^Ed^.

Data were collected from a total of 28 trials. All test were entirely conducted with no collisions or falls. As an illustration of the interaction with the high level of social engagement, Figure 3 depicts several moments of a trial with one of the users.

### 4.1 Illustrative case

Employing the sensor interface integrated into the Socially Assistive Walker (SAW), the platform operator was able to gather environmental, kinematic, and kinetic indicators from the user. The operator used this information to assess the system states and properly trigger the feedback behaviours. The following results showcase the physical interaction parameters from a single user to demonstrate the outputs of the sensor interface.

**FIGURE 4:**
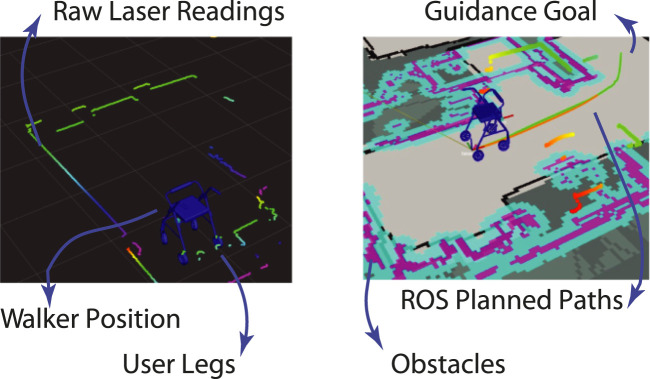
Illustration of environment information used by the ROS navigation stack. **(A)** Raw acquisitions from the laser scanners. **(B)** Processed obstacles and generated paths.

On the one hand, to maintain users’ safety, collect environmental data related to the walker’s position within the experimental setup, provide safe guiding, and track users’ motion, the interface sensors fed the ROS navigation stack to process laser scanners data into inflated obstacles, plan paths or routes within the experimental setup, and provide autonomous localisation. Figure 4 illustrates the presented information to the external operator.

**FIGURE 5:**
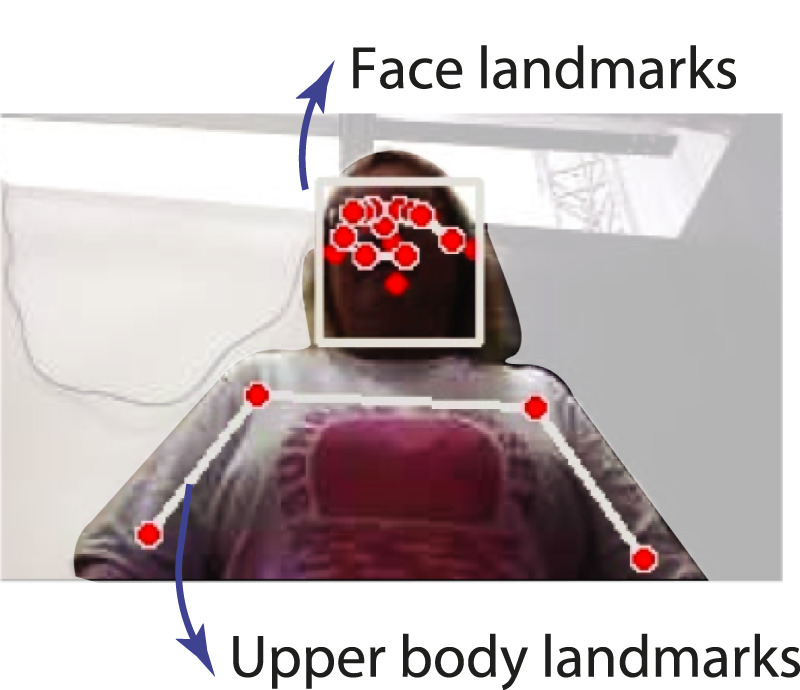
Illustration of posture tracking by Google’s Mediapipe pre-trained model.

On the other hand, to track users’ posture during sit-to-stand and stand-to-sit transfers, as well as to trigger information cues during the food reminder task, the device’s camera was used to track the upper body of the user using Google’s mediapipe solutions (Google Developers Team, 2023). Figure 5 illustrates the annotated image obtained after passing the camera video stream through the mediapipe’s posture model. This information allowed the external operator to track whether the user was looking at the screen or not, the exact moment when the user follow posture corrections, as well as sitting and standing instructions.

**FIGURE 6:**
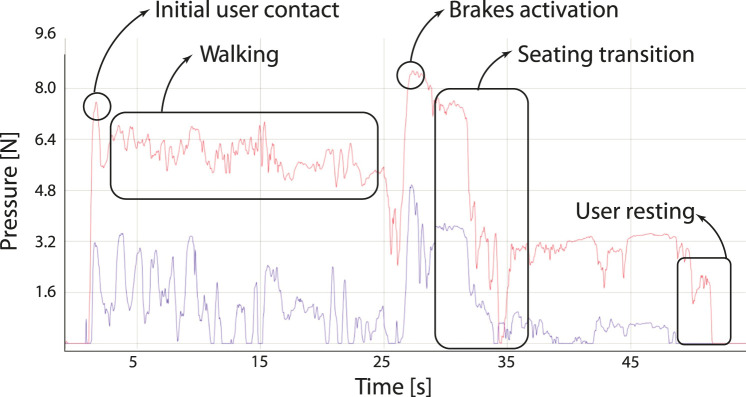
Illustration of the sit-to-stand task and the acquired signals with FSRs placed on the walker’s handles. The left signal is illustrated in blue colour, and the right signal in red colour.

Regarding physical interaction, the sensors on the handles provide pressure information about the user’s gripping. The collected data with the Force Resistive Sensors (FSRS) was used to track the user’s walking pattern, brake activation, and weight support on the frame. Figure 6 illustrates the acquired signals between the session’s start and the stand-to-sit task’s end. Several states are indicated according to the behaviour of the right handle signal.

### 4.2 Qualitative measurements

The validation group was conformed by 14 volunteers without gait assistance requirements or cognitive disorders (11 males, three females, 51.7±6.6 y.o., 1.73±0.07 m, 77.6±14.6 kg), and their data is summarised in Table 3. The validation group targeted healthy adults to validate the prototype and the perception surveys in users without cognitive impairments in an environment with reduced risk conditions. The age range (i.e., older than 40) was selected to match the average age of caregivers in assisted living scenarios. As stated in Table 3, the validation group is highly comfortable with technology, familiar with robotic walkers or similar devices, and primarily not interested in using robotic walkers at the time of the study, as expected.

**TABLE 3:** Demographic data of the participants.

Educational Level	
- Postgraduate	71.43%
- Bachelor	14.29%
- Diploma	7.14%
Occupation	
- University Researcher	50%
- Business Coordinator	7.14%
- Administrative	14.28%
- Technician	14.28%
- Director of Teaching	7.14%
- Nursery Nurse	7.14%
Living Arrangement	
- Alone	28.57%
- With Family	35.71%
- With Partner	35.71%
Familiar to Robotic Walkers or Similar	
- Seen or heard about them	69.23%
- Unfamiliar	30.77%
Comfort with Technology	
- Very Comfortable	57.14%
- Comfortable	35.71%
- Neutral	7.14%
Interested in Using a Robotic Walker	
- Not at all	50%
- No, but open to it	28.57%
- Not sure	7.14%
- Yes, maybe	7.14%

#### 4.2.1 Perception questionnaires

**FIGURE 7:**
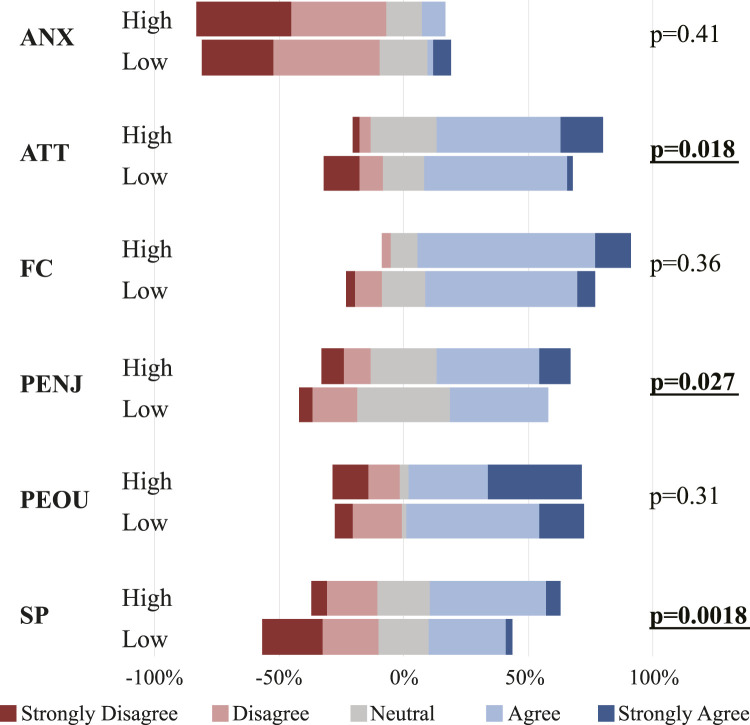
Distribution of the answers of the qualitative questionnaire by interaction mode, i.e., high vs. low. Anxiety (ANX). Attitude (ATT). Facilitating Conditions (FC). Perceived Enjoyment (PENJ). Perceived Ease of Use (PEOU). Social Presence (SP). *p-values* in bold indicate significant differences between high and low interaction modes.

Regarding the perception results, Figure 7 illustrates the response distribution for the social engagement levels at each qualitative category. Similarly, the *p-value* is shown, highlighting the categories with significant differences between social engagement levels. The Anxiety (ANX) questions aimed to assess the perceived fear of breaking or making mistakes with the device. The answers for this category were mainly negative, and no significant differences were found. This result is expected since the device’s design followed recommendations and insights from the semi-structured interviews with healthcare experts to ensure it is perceived as a non-complex technology. Regarding the Attitude (ATT) category, primarily positive answers were obtained for both social engagement levels. However, significant differences were received, with the high level having a slightly more positive distribution. In general, these questions sought to determine if the particular version of the robotic walker could be useful in improving daily living activities. Thus, this result might be driven by the embodiment and verbal cues programmed in the high level of interaction.

Regarding the Facilitating Conditions (FC), such questions evaluated whether the users understood and had enough training to properly use the robotic walker. In this case, responses with predominant positive distributions were obtained, and no significant differences were found. This outcome suggests that despite the interaction level, the users could properly understand the instructions and communication from the device. In regards to the Perceived Enjoyment (PENJ), this category was aimed at determining if the users enjoyed the interaction channels employed by the device under each level of interaction. Interestingly, positive distributions were obtained for the two levels of interaction, and significant differences were found. In particular, the high level of social engagement exhibited a more positive distribution, suggesting that the participants showed a greater preference and enjoyment for it compared to the low level of interaction.

Looking at the Perceived Ease of Use (PEOU), the proposed questions sought to assess intuitiveness, interaction naturalness (i.e., simple communication), and technological simplicity. In this case, both levels of interaction showed positive distributions with no significant differences between them. This result could be attributed to the overall design of the social robotic walker, which is perceived as an easy-to-use device regardless of the feedback or interaction cues. Finally, the Social Presence (SP) category was designed to identify whether the users perceived differences in terms of being assisted by *real* agent, the preference for the communication strategies, the number of social interaction skills, and the perception of the device as an intelligent agent. In this case, more positive responses were obtained under the high level of interaction, with significant differences between the levels. These results suggest that users were able to identify the most significant social presence with the high mode, and their preference was shifted to it.

#### 4.2.2 Final and open-ended questionnaire

Considering that this study was conducted with healthy adults, one of the objectives of the final questionnaire (i.e., applied after experiencing both levels) was to determine the expected potential and applicability of the proposed device in older adults with physical or cognitive impairments. It is important to acknowledge that 50% of participants expressed no interest in using a robotic walker, as indicated in Table 3. This lack of interest may introduce a bias in the data, as it reflects the views of a subset of participants who do not see the immediate need for such a device. This perception likely stems from the fact that these participants do not currently require a robotic walker to carry out their daily activities. However, this does not necessarily reflect the overall efficacy or necessity of the device. Particularly,^R1^ 93% of the participants stated that the proposed solution would be able to assist the activities of daily living to older adults, of which 100% mentioned that at least one older relative (e.g., parents) could benefit from the social robotic walker at the time of the study.

**FIGURE 8:**
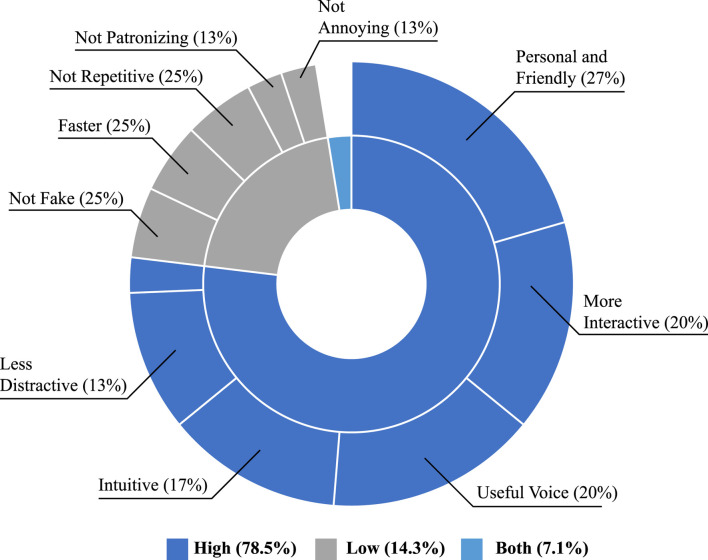
Perceived qualitative attributes and users’ preference. Inner ring shows the percentage of users that preferred each interaction level. Outer ring shows the perceived attributes by the users who preferred each mode.

Furthermore, to validate the findings of the perception questionnaires, the participants were asked if they noticed differences between the trials. In this case, all of the users noticed differences, and the 78% preferred the high interaction level, the 14% preferred the low interaction level, and the 7% suggested a combination of them. Regarding the attributes perceived by the participants, Figure 8 illustrates those assigned to each mode when the users preferred each mode. As shown in the figure, the attributes underscored for the high level of interaction were primarily positive, stating its friendliness, intuitive interaction, and the usefulness of the embodiment (e.g., face and voice). Similarly, the participants noticed that the high level of interaction was less distracting due to not needing to read texts from the tablet and the location of it. Regarding the low level of interactions, the users that preferred this mode highlighted that it was faster, not repetitive or annoying, and particularly not fake. The users mentioned that the high mode tended to overreact or to be excessively encouraging during simple tasks.

Finally, participants’ feedback suggested several potential enhancements, removals, and modifications to the walker’s design. To enhance user experience, it was recommended to add features such as suspension for navigating small bumps, combining modes for improved usability, providing detailed information about braking and real-time notifications, incorporating haptic feedback and collision warnings, and enabling automatic brakes during fast walking or sit-to-stand transitions. Additionally, participants preferred more interactivity, biofeedback for heart rate, lights for nighttime guidance, and the ability to play music and count steps. On the other hand, to streamline the design, participants suggested removing features such as the camera to reduce intrusiveness, avoid chirpy voice tones, remove repetitive prompts or notification sounds, and modulate flashing lights, considering potential sensitivities. The suggested modifications include adjusting the screen placement for safety, managing LED brightness, raising indicators for better visibility, offering a variety of verbal expressions, and providing customisation in terms of voices, language, humour level, colours, and features. These insights provide valuable input for refining the walker’s design to align more closely with user preferences and needs.

### 4.3 Limitations

Despite the promising results, several limitations should be acknowledged. First, the study’s sample size was relatively small, consisting of 14 participants. While this sample size was adequate for qualitative insights and initial feedback, larger-scale studies are needed to confirm the generalisation of these findings across broader populations and diverse geographical regions. Second, the study primarily focused on healthcare professionals and experts in geriatric care. While their insights were valuable for informing the design criteria of the SAW, future research should also include direct input from older adults themselves and their caregivers to ensure the device meets their specific needs and preferences. Third, the study duration was limited, and longer-term assessments are needed to evaluate the sustained usability and acceptance of the SAW over extended periods. Longitudinal studies would provide valuable insights into how user interactions with the device evolve over time and its impact on their daily lives.^R1^ Fourth, rollator users sometimes have visual impairments or physical limitations, which can affect their ability to control the walker and may lead to collisions, damaging components such as tablets, speakers, or cameras. It is important to note that this study presents a first prototype of a socially assistive walker. As such, the current prototype may not fully address all challenges faced by users with diverse needs and abilities. Future iterations of the prototype will focus on enhancing user interaction design, integrating robust collision avoidance mechanisms, and ensuring the durability of external components.^R2^

## 5 Conclusions and future works

This study collected data from adults interacting with a Socially Assistive Walker (SAW) during daily living activities. The device was designed based on the findings of semi-structured interviews with ageing experts, and two levels of social engagement or interaction were designed to assess users’ preferences. The high level of social engagement demonstrated its effectiveness in interaction, as illustrated in Figure 7, being preferred by most users. The validation group provided valuable insights regarding future modifications of the device’s design. These mainly focused on tailoring the interaction by providing customisation options to the virtual agent regarding voice tones, language tone, feedback modes, and appearance.

Furthermore, given that one of the design criteria that resulted from the interviews was avoiding over-complex interactions, automatic brakes, haptic feedback, and speech interaction were not included in this device version. However, the users suggested adding these features with the possibility of configuring them to meet specific user needs. In terms of the potential impact on the target population, even though they were particularly interested in using them (i.e., healthy adults), they found the device easy to use regardless of the interaction mode. They suggested its usefulness for older adults with physical and cognitive impairments like dementia.

Future work will assess the suggestions for improvement collected from the current study, and the obtained results will be re-evaluated by ageing experts. The next version of the robotic device will be redesigned bearing in mind these findings, and it will be deployed for mid- to long-term assessment in care homes or residential settings. Moreover, analysing which pathologies could benefit from features like integrated tablets or LED rings is essential to assess usability, safety, and impact on fall risk. Future research should prioritise testing and validating the SAW features with diverse pathologies to understand their real contributions.^R2^

Future research will also focus on assessing the familiarity and long-term usability of the selected SAW among potential users. Recognising that acceptance and positive feelings towards assistive technologies often improve with prolonged exposure, we propose extended studies where users interact with the SAW in their daily lives over several months. This will help to assess how familiarity with the device affects user acceptance and overall satisfaction. Additionally, future work will evaluate the effectiveness of the SAW’s sensors and feedback mechanisms in real-world settings, monitoring usage patterns and gathering feedback on daily interactions to optimise the device further.^R1^

## Data Availability

The original contributions presented in the study are publicly available. This data can be found here: https://doi.org/10.6084/m9.figshare.25393525.v1
